# A Rare Case of Extramedullary T/Myeloid Mixed Phenotype Acute Leukemia with t(1;5)(q23;q33)

**DOI:** 10.1155/2016/8937940

**Published:** 2016-12-26

**Authors:** Ahmad Monabati, Akbar Safaei, Sadat Nouri, Moeinadin Safavi, Freidoon Solhjoo

**Affiliations:** Department of Pathology, School of Medicine, Shiraz University of Medical Sciences, Shiraz, Iran

## Abstract

Mixed phenotype acute leukemia (MPAL) is a rare neoplasm which accounts for 2–5% of all leukemias and it is classified under heading of acute leukemia of ambiguous lineage in 2008 WHO classification. This patient was a 61-year-old man who presented with malaise and weakness. In physical examination there was cervical and axillary lymphadenopathy. Paraclinical evaluation revealed anemia (Hb = 10.3 g/dL, MCV = 108 fl). Histologic sections of the axillary lymph node revealed leukemic involvement with two discrete populations of cells in immunohistochemistry. One population was immunoreactive for MPO and the other showed immunostaining for CD3, CD99, and tdt. Differential count of bone marrow cells in marrow aspirate had 6% blast. Karyotype study on bone marrow culture depicted an interesting finding which was t(1;5)(q23;q33). An extensive search on literature was done for the same genetic change. A similar translocation has been mentioned in literature for other hematologic malignancies but not for same neoplasm; anyhow this translocation was an imbalanced one and led to der(5)t(1;5)(q12-25;q13-q35).

## 1. Introduction

Mixed phenotype acute leukemia, according to the WHO 2008 classification, is a category of acute leukemia with ambiguous lineage (another category is acute undifferentiated leukemia) and is divided into acute bilineage (or multilineage) leukemia and acute biphenotypic (multiphenotypic) leukemia. While the former has more than one population of blast cells and total number of blasts from both lineages is more than 20% of marrow or blood cells, in the latter, one population of leukemic cells coexpress more than one lineage-specific marker.

The lineage-specific markers used for lineage assignments in acute leukemias are as follows: myeloid differentiation is based on expression of myeloperoxidase (MPO); at least two of the following markers: NSE, lysozyme, CD11c, CD14, and CD64, are used for monocytic differentiation; strong expression of CD19 with at least one of the following markers: CD79a, cytoplasmic CD22, and CD10 or weak CD19 expression with strong coexpression of at least two of the following markers: CD79a, cytoplasmic CD22, and CD10 is indicator of B-cell lineage; cytoplasmic or surface CD3 is specific for T-cell lineage [1, 2].

## 2. Case Presentation

**Figure 1:**
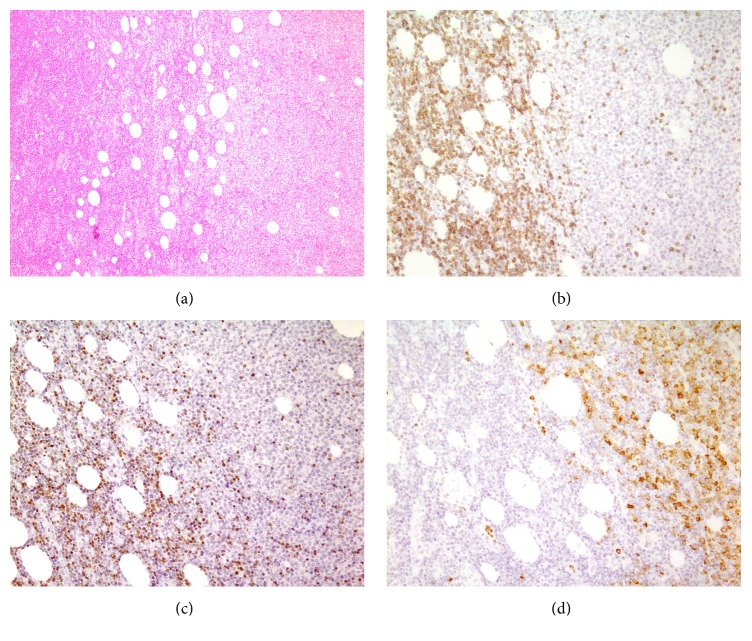
(a) Histologic section of lymph node showed effaced architecture by infiltration of bipopulation of blastoid cells (H&E stain ×100). (b) Positive immunostaining for CD3 in one population of blastoid cells (counterstain with hematoxylin ×400). (c) Positive immunostaining for tdt in the same population of blastoid cells (counterstain with hematoxylin ×400). (d) Positive immunostaining for MPO in another population of blastoid cells (counterstain with hematoxylin ×400).

**Figure 2:**
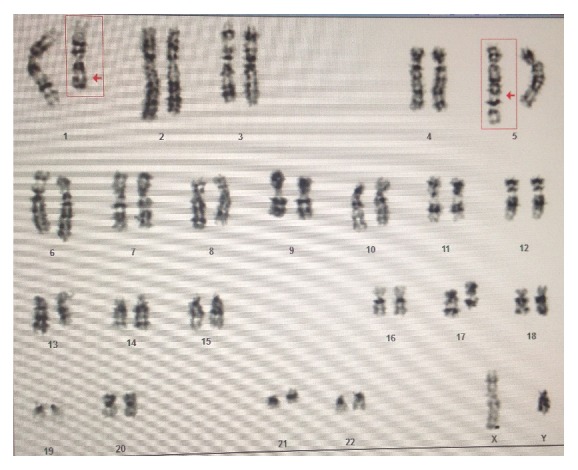
Karyotype analysis of the bone marrow culture revealing t(1;5)(q23;q33).

Patient was a 61-year-old man who denied any significant past medical history and presented with malaise and weakness. In physical examination lymph nodes enlargement in neck and axilla was found. Paraclinical evaluation was done and revealed anemia with anisopoikilocytosis (Hb = 10.3 g/dL, MCV = 108 fl), moderate leukocytosis, and about 3% immature cells. Histologic section of excised axillary lymph node showed two separate blast populations. In immunohistochemistry one population was immunoreactive for MPO and CD68 and negative for CD10, CD3, CD20, CD56, CD34, CD138, CD117, tdt, CD99, and S100, and the other showed expression of CD3, CD99, and tdt but not MPO, CD10, CD20, CD56, CD34, CD138, and CD117 (Figures 1(a)–1(d)). In both populations Ki67 highlighted about 70% of the cells. Bone marrow aspiration exhibited hypercellularity with orderly erythroid maturation and increased myeloid to erythroid ratio. About 6 percent immature cells in bone marrow aspiration smears were counted and the exact lineage of blasts in the marrow was not determined. Cytogenetic study on metaphase cells by Q-banding method depicted an interesting finding which was t(1;5)(q23;q33) (Figure 2).

## 3. Discussion

Mixed phenotype acute leukemia (MPAL) is a rare neoplasm which accounts for 2–5% of all leukemia [3, 4] and extramedullary presentation of them is extremely uncommon. Here we introduce a case of T/Myeloid mixed phenotype acute leukemia presented in lymph node which has t(1;5)(q23;q33). This is an unusual presentation of a rare disease with a chromosomal rearrangement not recorded previously [5]. In some studies, t(1;5)(q23;q33) is associated with myeloproliferative disorder with eosinophilia [6, 7], but this patient has no concomitant eosinophilia or previous history of myeloproliferative disorder. Also this chromosomal rearrangement was reported in association with B-lineage acute lymphoblastic leukemia [8]. A similar translocation has also been reported in mixed phenotype acute leukemia, B/Myeloid, NOS; plasma cell neoplasm; diffuse large B-cell lymphoma (DLBCL), NOS; primary DLBCL of the CNS; primary cutaneous DLBCL, leg type; EBV positive DLBCL of the elderly; DLBCL associated with chronic inflammation; B-cell lymphoma, unclassifiable, with features intermediate between DLBCL and Burkitt lymphoma; hepatosplenic T-cell lymphoma; mycosis fungoides and follicular lymphoma; however this translocation is usually imbalanced and led to der(5)t(1;5)(q12-25;q13-q35) [5]. As mixed phenotype acute leukemia is a rare and a heterogenous disorder [9, 10], adequate information concerning clinical and biologic aspects of this type of malignancy is not present [4]. We believe that clinical and cytogenetic findings in this case are quite interesting and merit attention.
